# Isolation and Characterization of a Novel Diethylstilbestrol-Degrading *Bacillus subtilis* JF and Biochemical Degradation Metabolite Analysis

**DOI:** 10.3389/fmicb.2019.02538

**Published:** 2019-11-08

**Authors:** Weiqin Deng, Yun Zhao, Kaidi Hu, Shujuan Chen, Li He, Xiaolin Ao, Likou Zou, Xinjie Hu, Yong Yang, Shuliang Liu

**Affiliations:** ^1^College of Food Science, Sichuan Agricultural University, Ya’an, China; ^2^Sichuan Institute of Research and Design About Food and Fermentation Industries, Chengdu, China; ^3^Institute of Food Processing and Safety, Sichuan Agricultural University, Ya’an, China; ^4^College of Resources, Sichuan Agricultural University, Chengdu, China

**Keywords:** diethylstilbestrol, *Bacillus subtilis*, biochemical degradation, metabolite, pathway

## Abstract

Diethylstilbestrol (DES) can adversely affect the immune system of developing fetuses or even elicit toxic responses such as nerve toxicity and genotoxicity in human beings, thereby warranting methods to remove DES from the environments. The present study characterized a novel DES-degrading *Bacillus subtilis* JF and analyzed the degradation metabolites. The strain was collected at the China General Microbiological Culture Collection Center (Collection number: CGMCC 7950). The environmental effects, such as DES concentrations, pH levels, and temperature, on the strain’s degradation ability were tested. Degradation metabolites of DES by strain JF were analyzed via high performance liquid chromatography (HPLC) and liquid-chromatography time of flight mass spectrometry (LC-TOF-MS). Results indicated that *B. subtilis* JF can effectively degrade DES within a concentration of 25–200 mg/L. Increasing pH levels (pH > 7) are reported to increase the degradation rate of DES by the strain. The optimal temperature for strain JF to degrade DES was identified as 45°C. In this study, 4, 4′-hexene estrogen quinones (DESQ) and DES-4-semiquinone were speculated as two degradation metabolites of DES, and both can be completely degraded by strain JF. A slight reduction of DES in the blank system [DES cultured in Luria-Bertani (LB) medium without strain JF] was observed in this study. The reduction trend in the blank system only occurred during the first few days (about 4 days) and was considerably lesser than the decomposition and transformation effect of DES via strain JF. Furthermore, the metabolite DESQ could not be further decomposed in blank LB medium without strain JF. All the results demonstrate that complete degradation of DES in the fermentation broth occurs due to the function of strain JF rather than organic decomposition. In conclusion, the high efficiency of degradation and the potential to degrade DES completely indicates that strain JF has potential for the bioremediation of DES-contaminated environments (soil, river, and so on) and fermented foods.

## Graphical Abstract

Biodegradation of DES contamination.

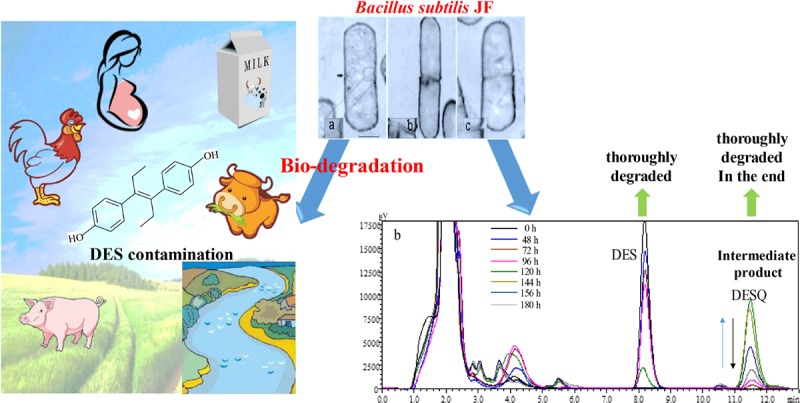

## Introduction

Diethylstilbestrol (DES) is a synthetic non-steroidal estrogen, previously used to prevent habitual abortion in pregnant women from 1940 to 1970 in the United States. However, despite its positive effect in preventing habitual abortion or even premature birth in expectant mothers, further research revealed that there would be higher incidence of abortion and premature birth when women were exposed to DES (Dieckmann et al., 1953; Brackbill and Berendes, 1978). Due to its highly toxic nature, the U.S. Food & Drug Administration has issued severe warnings about the harmful effects of DES and has released regulations prohibiting the use of DES during pregnancy (Food Drug Administration, 1971). In contrast, few studies reported that DES can improve the growth rate of animals and increase the protein content as it was once used to promote growth in livestock and aquaculture (Du and Xu, 2000; Liu et al., 2008); however, subsequent researches identified that the accumulation of DES in animal products such as liver, eggs, and milk, which are consumed by expectant mothers, may lead to crucial endocrine disorders and obesity in the offspring (Newbold et al., 2009). Moreover, DES affects the immune system of developing fetuses (Giusti et al., 1995), and in some cases, it can even elicit a toxic response such as neurotoxicity (Ryan and Vandenbergh, 2006) or genotoxicity (Rissman and Adli, 2014; Lei et al., 2016). Furthermore, long-term intake of DES in limited concentration may lead to endocrine disorders and increase the incidence of cancer (Lucci et al., 2011). For instance, 2 g of DES accumulated in the human body led to breast cancer (Giusti et al., 1995). DES has gained immense social attention, and several countries have banned the use of DES as a growth promoter due to its toxic effects (Judson et al., 2009).

Although DES has been banned in clinical treatment and animal production, in several countries, its traces are still present in the river waters and livestock milk and meat (Bravo et al., 2007; Xiong et al., 2014; Yin et al., 2016). Yin et al. (2016) detected DES in seawater, shrimp, and fish, with DES residues as 2–8, 1.86–7.69, and 2.13–8.28 μmol/L, respectively. Nazli et al. (2005) randomly collected 60 meat samples in Istanbul, Turkey, amongst which 35% were detected as DES positive and 15% were even found to possess more than 1 μg/kg DES. Similarly, another research group (Solé et al., 2000) studied the DES contamination in sewage treatment plants in Catalonian, Spain. Their findings revealed that sewage effluent contained 34 ng/L of DES. The DES residue has a great potential to re-endanger the human population via environmental pollution and food chain contamination (Korshin et al., 2006).

Therefore, research on the degradation or elimination of DES from the environment and animal food products is essential for the well-being of human population. Biodegradation is an ideal low-cost approach for eliminating contaminants (Chen et al., 2010) rather than generating secondary pollutants. Presently, few studies are targeting biodegradation of DES, in particular, only two microorganisms have ever been reported to harbor the capability to degrade DES. Zhang et al. (2013) screened a *Pseudomonas* sp. J51 strain with DES degradation ability. Xu et al. (2014) has obtained a DES-degrading strain S from the activated sludge of a sewage treatment station. The strain was identified as *Serratia* sp., and it can degrade 68.3% of 50 mg/L DES within 7 days. These degradation rates are not rapid enough to eliminate DES. Therefore, the screening of microbial strains with high DES degradability is necessary. Moreover, it is essential to determine whether the by-products that completely degrade DES are toxic or non-toxic.

It is necessary to explore the pathway and mechanism of the DES biodegradation. Few studies have reported the mechanism of DES degraded through silver oxide (Liehr et al., 1983), wherein peroxidase-catalysation action (Metzler, 1984) and photo-oxidation (Zhou et al., 2004) were observed. DES-4-semiquinone and DESQ were identified as the intermediates produced via DES degradation. In this research, a DES-degrading strain *Bacillus subtilis* JF and the degrading characteristic of DES by the strain in different conditions, such as substrate concentration, initial pH, and culture temperature were studied. Thereafter, the subsequent metabolites and pathways of DES degradation were investigated. Thus, strain JF can be applied in the biodegradation process of DES in contaminated foods and environment.

## Materials and Methods

### Chemicals

Diethylstilbestrol (99.5% purity) was purchased from Merck KGaA, Germany. HPLC-grade acetonitrile was purchased from CNW Technologies GmbH-Dusseldorf, Germany. HPLC-grade methanol was purchased from Guangdong Guanghua, Co., Ltd., China.

### Microorganisms and Media

#### Media

Mineral salt medium (MSM), comprising 1.5 g of (NH_4_)_2_SO_4_, 0.5 g of K_2_HPO_4_, 1.5 g of KH_2_PO_4_, 0.2 g of MgSO_4_, and 0.5 g of NaCl per 1000 mL of distilled water, was used. The solution was adjusted to pH 7.0 and sterilized at 121°C for 20 min.

Luria-Bertani (LB) medium containing 5.0 g yeast extract, 10.0 g peptone and 10.0 g NaCl per 1000 mL was used and sterilized at 121°C for 20 min.

Normal saline comprising 0.85 g NaCl per 100 mL of distilled water was used and sterilized at 121°C for 20 min.

### DES Stock Solution

Diethylstilbestrol was dissolved in ethanol to prepare the DES stock solution, with a concentration of 10 mg/mL.

### Enrichment, Isolation, and Screening of the DES-Degrading Strain

The vegetable field soil and waste water from Ya’an, Sichuan Province of China, were used for isolation of DES-degrading strain. The sample (5 g) was transferred into a 250 mL Erlenmeyer flask containing 30 mL LB and 20 mg/L DES, and was then incubated at 37°C in an oscillating incubator at 140 rpm for 48 h. The strain was successively transferred to LB media containing 20 mg/L of DES for purification. These strains were inoculated on the LB slants, cultured for 24 h, and then washed with sterile saline (5 mL) solution to obtain the inoculate. Inocula was transferred (1 mL) to 29 mL of fresh LB containing 20 mg/L DES, with a concentration of about 10^7^ CFU/mL; the control was inoculated with the same amount of sterile saline solution and incubated for 72 h. The concentrations of DES residues in each culture were detected via high-performance liquid chromatography (HPLC, LC-10A2010C HT, Shimadzu-Japan) according to the methods of Deng et al. (2016).

Final cultures of strains that could effectively degrade DES were spread on the LB plates containing 20 mg/L of DES and were incubated for 72 h. The inocula were prepared by washing the bacterium lawn and adjusting with sterile saline solution, thereby achieving a density of 10^9^ CFU/mL. The inocula were transferred (1 mL) to 29 mL of fresh LB containing 20 mg/L DES; the control was prepared with the same amount of sterile saline solution. The concentrations of DES residues in various cultures were determined via HPLC and the strain that revealed highest degradation ability was selected for the succeeding experiments.

### Identification of the Strain JF

The JF strain was identified and characterized using biochemical methods in combination with 16S rRNA gene sequence analysis. Biochemical identification was achieved according to eighth edition of Bergey’s Manual of Determinative Bacteriology. Colony morphology of the strain JF was documented after inoculation on LB plates, and these culture plates were then incubated at 35°C for 48 h. Thereafter, Gram’s staining was performed with a single colony and the slides were observed with an Olympus BH-2 light microscope (Olympus-Tokyo, Japan). The cell form of strain JF was detected by Apreo SEM (Thermo Fisher Scientific, United States).

Total genomic DNA was extracted with the DNA extraction kits (Tianze Genetic Engineering Co., Ltd-Dalian, China). The 16S rRNA sequence was amplified by polymerase chain reaction (PCR; C1000 Thermal Cycler, Bio-Rad, United States) with the universal primers 27F (5′-AGAGTTTGATCC TGGCTCAG-3′) and 1492R (5′- GGTTACCTTGTTACGACTT-3′) produced by Biological Engineering Co., Ltd-Dalian, China. T carrier cloning and sequencing of the cloned insert were performed by Biological Engineering Co., Ltd-Dalian. The 16S rRNA sequence was compared by BLAST search through the National Center for Biotechnology Information (NCBI) website.

### Bio-Degrading Characteristic of DES by Strain JF

In order to study the degradation characteristics of DES by strain JF, the impact of DES on the growth of strain JF and the effect of DES concentration, initial pH of medium and different culture temperature on the degradation ability of strain JF were studied, respectively, in this research.

### Influence of DES on the Growth of the Strain JF

The *B. subtilis* JF strain was cultured on an LB agar slant containing 50 mg/L of DES, and the bacterium lawn was washed with 5 mL sterile saline solution to obtain the inocula. About 1 mL inocula was transferred into 29 mL fresh LB (pH 7.0) in an Erlenmeyer flask, with the initial cell concentration of about 10^7^ CFU/mL. DES and 0.2% Tween 80 were added to obtain a final concentration of 100 mg/L. A cultivate system added equal volume of ethanol (about 1%, v/v) instead of DES was set as control. Thereafter, the culture medium was incubated at 40°C in an oscillating incubator at 140 rpm. The samples were extracted at 12, 24, 48, 72, and 96 h, in order to detect the biomass of JF. Biomass was measured using the UV VIS spectrophotometer (UV-3200, PC Shanghai Meipuda Instrument Co., Ltd.) at an absorbance of 600 nm. Triplicate samples were extracted and analyzed.

### Influence of DES Concentration on Strain JF Degradability

The inocula were transferred into fresh LB (pH 7.0) medium by adding DES to achieve a concentration of 25, 50, 100, and 200 mg/L, respectively. About 30 mL of inocula were separated into different Erlenmeyer flasks. Next, these were incubated at 40°C in an oscillating incubator at 140 rpm. Meanwhile, the cultivate system containing an equal volume of sterile saline solution instead of strain JF was set as control. Three replicates were set up. All samples were extracted daily and the concentration of DES in each culture was detected by HPLC (LC-10A2010C HT, Shimadzu-Japan) according to the methodology of Deng et al. (2016). Thus, the biodegradation rate of DES was calculated.

### Influence of Initial pH of Medium on Strain JF Degradability

The inocula were transferred into fresh LB medium at different pH levels (pH 5, 6, 7, 8, and 9). DES was added to obtain a DES concentration of 100 mg/L. Thereafter, the inoculation was separated into different Erlenmeyer flasks (30 mL per flask). Control cultivate system was set up with equal volumes of sterile saline solution instead of strain JF. Three experimental replicates were set up, all of which were incubated at 40°C in an oscillating incubator at 140 rpm. The concentration of DES in the culture was detected daily.

### Effect of Varying Culture Temperature on Strain JF Degradability

The inocula were transferred into fresh LB medium (pH 7.0), and DES was added to obtain a concentration of 100 mg/L. The culture was then separated into different Erlenmeyer flasks (30 mL per flask). Control cultivate system was set up with equal volumes of sterile saline solution instead of strain JF. Three replicates were set for each incubation. The culture was cultivated in an oscillating incubator at 140 rpm at different temperatures (30°C, 35°C, 40°C, and 45°C), respectively. The DES concentration in the culture was detected daily, according to the method described previously.

### Application of Strain JF

Considering that DES was mostly detected in the sewage and meat products, we tried to use strain JF to treat the river water and pork (DES was added).

The DES was transferred into sterilized river water (30 mL in Erlenmeyer flask) to 2 mg/L, then strain JF was inoculated to 10^7^ CFU/mL. Another system comprising an equal volume of sterile saline solution instead of strain JF was set as the control. Three replicates were set up. Samples were incubated at 40°C in the oscillating incubator at 140 rpm for 14 days. The concentration of DES in the culture was extracted and measured via HPLC.

About 100 g minced meat (pork, fat removed) was added into a 250 mL Erlenmeyer flask, and was sterilized at 121°C for 20 min. DES was added up to a concentration of 2 mg/L, followed by JF strain up to 10^7^ CFU/mL. Another system containing equal volume of sterile saline solution, instead of strain JF, was set as the control. Three replicates were set up. Samples were incubated at 40°C for 14 days. The concentration of DES in the culture was detected via HPLC.

### Identification of DES Metabolites Degraded by Strain JF via HPLC and LC-TOF-MS

Inocula were transferred into fresh LB (pH 7.0.) with DES (100 mg/L), and then sub-packaged in Erlenmeyer flasks (30 mL per bottle). A control cultivate system with an equal volume of ethanol, instead of DES, was simultaneously set up. Three experimental replicates were set up, respectively. All of the samples were cultured at 40°C in the oscillating incubator at 140 rpm. One whole bottle of culture sample was collected every 12 h. About 1 mL culture was collected for DES extraction, and the remaining samples were further stored at 4°C (for next step, LC-TOF-MS analysis). The concentration of DES residues in various cultures was determined via HPLC.

All samples collected from different cultivated time were mixed together and centrifuged at 8000 rpm for 10 min at 4°C to remove the bacteria and impurities. The supernatant was lyophilized by lyophilizer (Powerdry PL 3000, Thermo Fisher Scientific, United States) after pre-freezing at−20°C. The lyophilized powder was dissolved with 10 mL methanol, and further centrifuged at 12,000 rpm for 10 min. The supernatant was treated with activated carbon to deodorize and decolor. Thereafter, it was centrifuged at 12,000 rpm for 10 min, and then filtered with a 0.45 μm organic phase membrane filter. The filtrate was detected via LC-TOF-MS.

LC-TOF-MS investigations were carried out under the following conditions. Agilent 1200SL-HPLC, G6210A- LC-TOF/MS-Waters with Agilent ZORBAX SB-C_18_ (1.8 μm, 2.1 × 100 mm, day) chromatographic column was used as the detector. The mobile phase was formed by acetonitrile and 20 mmol/L ammonium formate aqueous solution. The column gradient program comprised 5% acetonitrile and 95% ammonium formate aqueous solution in the first 10 min. The percentage of acetonitrile increased from 5% to 95% within 25 min linearly (5% at 10 min to 20% at 12 min, 70% at 15 min, 95% at 25 min). Column temperature was maintained at 40°C; injection volume was 10.0 μL with a flow rate of 0.3 mL/min. DAD detector was used with the detection wavelength of 242 nm. The instrument was operated in anion (ESI-) mode and the ion source parameters were adjusted as follows: the drying gas temperature was 350°C, capillary voltage was 3500 V, drying gas flow rate was 10.0 L/min, the nebulizer pressure was 40 psi, and the fragmentor voltage was 120 V. The range of mass-to-charge ratio (m/z) was from 50 to 1200, and the collection rate was 1.0 spectra/s with centroid mode.

### Data Analysis

Degradation rate was calculated according to Eq. 1.

(1) $$Degradationpercentage\left(\%\right)=\frac{\left(C_{0}-C_{t}\right)}{C_{0}}\times 100\%$$

where *C*_*t*_ is the residual concentration of DES in the sample solution (mg/L) and *C*_0_ is the initial concentration of DES (mg/L) at time zero.

The precision of the method was expressed as the relative standard deviation (RSD) of triplicate measurements.

## Results

### Isolation and Screening of DES-Degrading Strains

*Bacillus* B-1 and JF isolated from solid samples revealed that degradation ability of DES. *Bacillus* B-1 can degrade DES weakly (only 5%), whereas JF can degrade 30% DES efficiently (20 mg/L). Thus, JF was selected for further experiments in this study.

### Identification and Morphological Characteristics of Strain JF

**FIGURE 1:**
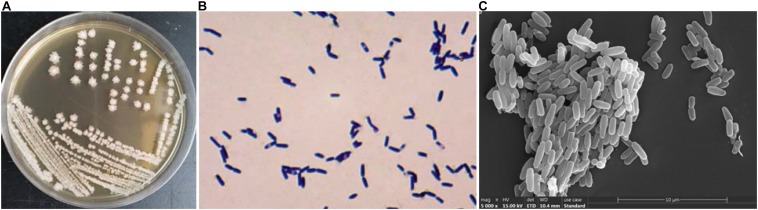
Characteristic of JF, **(A)** colony morphologies; **(B)** Gram staining micrograph; **(C)** SEM picture of JF.

Figure 1A depicts the strain JF colonies; they are flat, slightly white, and the periphery was yellowish; the surface was dry and wrinkled; and the edge was irregular. The cells of JF (Figure 1B) are rod-shaped, gram-positive, and produce central spores (spores are difficult to dye, and thus, they are transparent in the picture). Figure 1C is the SEM picture of JF, depicting rod-shaped cells and some germinating cells.

Biochemical characteristics of strain JF are presented in Table 1; the strain was aerobic, catalase positive, and phenylalanine dehydrogenase positive. It was able to hydrolyze gelatin and starch. It can metabolize glucose oxidatively and reduce nitrate. It can grow well in the condition containing 2–7% of NaCl but not with 10% NaCl. The strain can grow well at 30°C, 40°C and 50°C but not at 4°C or 60°C.

**TABLE 1:** Biochemical characteristics of strain JF.

**Characteristics**	**Response**	**Characteristics**	**Response**	**Characteristics**	**Response**
Anaerobic conditions	–	Hydrolysis of gelatin	+	Voges–Proskauer test	+
Acid production: D-glucose	+	Hydrolysis of starch	+	Nitrate reducing	+
L-arabinose	+	Growth- pH 6.8	+	Growth-temperature 4.0°C	–
D-xylose	+	Growth- pH 5.7	+	Growth-temperature 30°C	+
D-mannitol	+	Growth- NaCl: 2 g/100mL	+	Growth-temperature 40°C	+
Phenylalanine dehydrogenase	–	Growth- NaCl: 5 g/100mL	+	Growth-temperature 50°C	+
Yolk lecithin enzyme	–	Growth- NaCl: 7 g/100mL	+	Growth-temperature 60°C	–
Catalase	+	Growth- NaCl: 10 g/100mL	–	Growth-with muramidase	+

**FIGURE 2:**
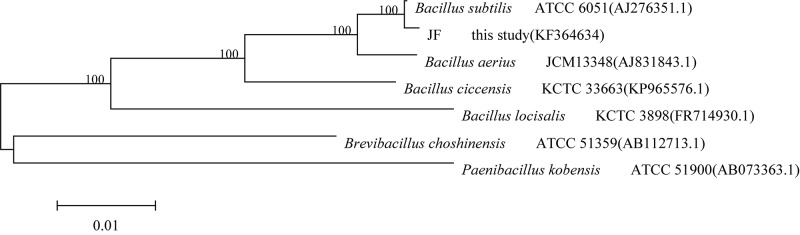
Phylogenetic tree based on the 16S rDNA gene sequences of DES-degrading strain JF.

The sequencing results revealed that the 16S rRNA gene fragment of strain JF was 1513 bp. It was registered in the GenBank database, and the accession number was KF364634. The sequencing results of the strain JF 16S rRNA gene were compared with the blast homology of the sequence in the GenBank database. A phylogenetic tree was constructed by the proximity method, as depicted in Figure 2. The 16S rRNA sequence of the strain JF was highly homologous with the gene sequence of type strain of *B. subtilis* ATCC 6051 (AJ276351.1); thus, strain JF was identified as *B. subtilis*.

### Growth Characteristics of *B. subtilis* JF

**FIGURE 3:**
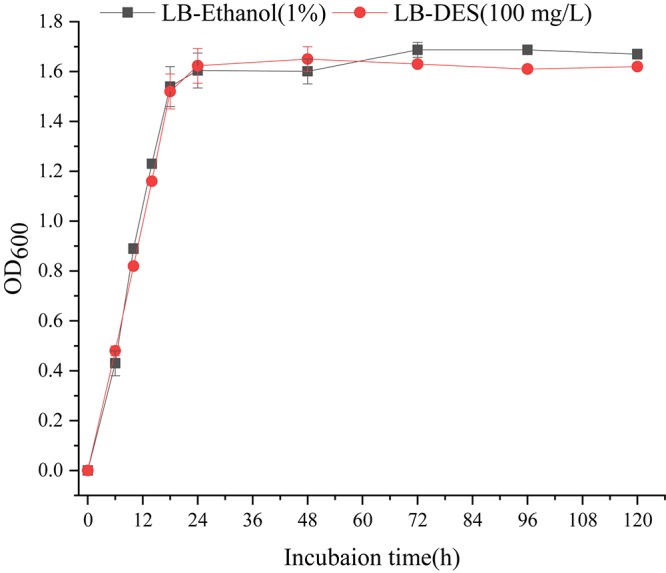
Effect of DES on the growth of JF.

The impact of DES on the growth of the strain JF was assessed based on the biomass (OD_600_) of JF cultivated in LB-DES (100 mg/L) medium. As DES was dissolved with ethanol, JF inoculated in LB medium with similar amount of ethanol (volume) was set as the control. Results are depicted in Figure 3. The growth pattern of the JF strain in LB-DES and LB-ethanol was essentially similar. The results revealed that DES did not significantly affect the growth of strain JF in the LB medium.

### Influence of DES Concentration on Strain JF Degradability

Strain JF’s degradability of DES in the MSM and LB medium was detected during commencement of the experiment. Results revealed that strain JF could degrade DES effectively in the LB medium but not in the MSM medium. This indicates that strain JF cannot use DES as a unique carbon source. The degradation of DES by strain JF is a co-metabolic degradation process, and a primary carbon source or other nutriments are required for the degradation (Zhang et al., 2017; Hazen, 2018). Thus, LB was selected as the basal culture medium for studying the degrading characteristic of DES by stain JF.

**FIGURE 4:**
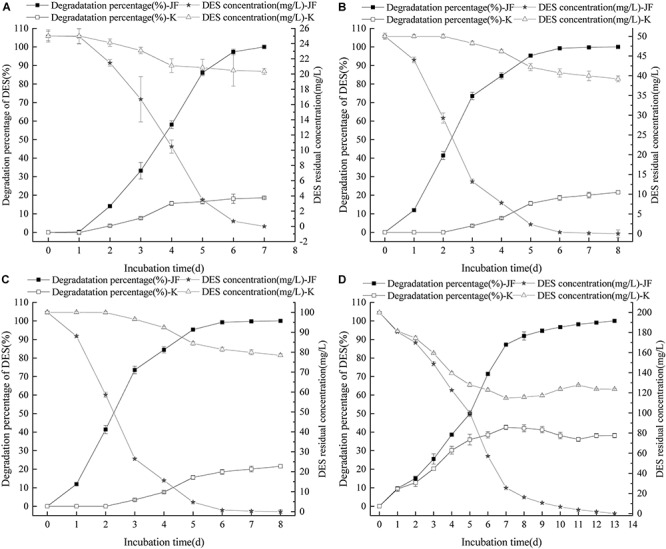
Effect of DES concentration on the DES degradability of JF **(A**: DES 25 mg/L; **B:** DES 50 mg/L; **C:** DES 100 mg/L; **D:** DES 200 mg/L). K is a control experiment, in which an equal volume of sterile saline solution was inoculated in the DES degradation system instead of strain JF.

The effects of different concentration of DES on the degradability of strain JF were tested. Results revealed that varying concentrations of DES in LB blank medium (control) had varying reduction rates as per Figure 4. Strain JF can effectively degrade DES from 25 to 200 mg/L. Unexpectedly, self-decomposition of DES in LB medium was detected. Strain JF can completely degrade 25 mg/L DES after culturing for 7 days, whereas 18.58% of DES in blank LB medium was reduced under identical conditions. About 50 mg/L DES can be totally degraded by strain JF after culturing for 8 days, but this reduced to 21.58% in blank LB medium under the same conditions. Moreover, 100 mg/L DES was degraded completely by JF in LB after being cultured for 11 days, but it reduced to 43% in blank LB medium under similar conditions; 200 mg/L DES can be degraded completely by JF in LB within 13 days, but it reduced by 38.19% in blank LB medium within 13 days. The degradation rate of DES in JF inoculated medium was much higher than the reduction rate in the blank LB medium. Figure 4 presents the reduction rates of DES gradually increasing only at the earlier stage (about 4 days), which were then stabilized in the cultivated stage. Thus, we can conclude that the total degradation of DES in fermentation broth is due to the action of strain JF rather than organic decomposition.

### Influence of Initial pH on Strain JF Degradability

**FIGURE 5:**
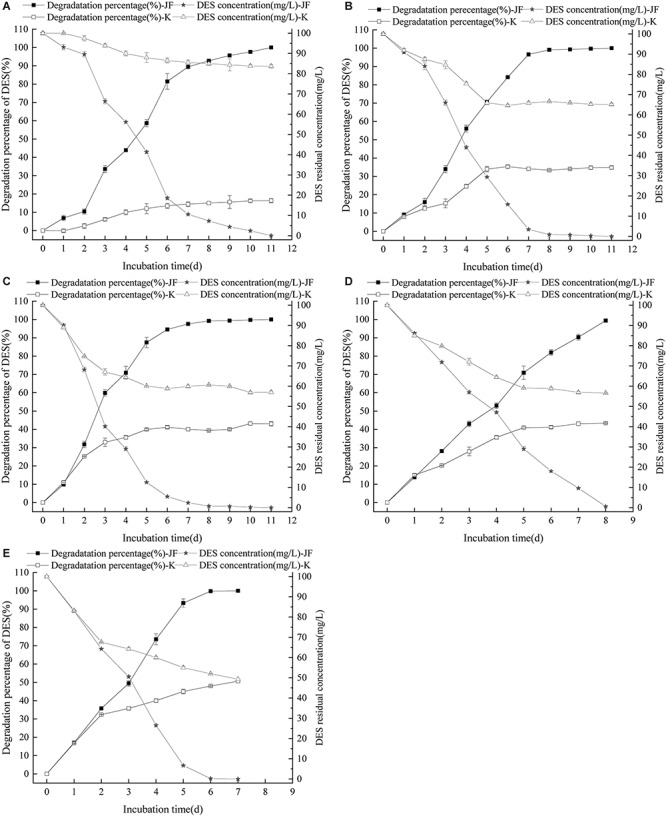
Effect of initial pH on the DES degradability of JF (**A:** pH 5; **B:** pH 6; **C:** pH 7; **D:** pH 8; **E:** pH 9). K is a control experiment, in which an equal volume of sterile saline solution was inoculated in the DES degradation system instead of strain JF.

Diethylstilbestrol can be degraded effectively by strain JF at different initial pH conditions as presented in Figure 5. Strain JF can effectively degrade DES at an initial pH of 5–9, but a significant difference in the reduction rates of DES in blank LB medium with different initial pH was noticed. When the initial pH value is 5, 100 mg/L DES can be completely degraded by strain JF within 11 days, and the automatic reduction amount of DES under the similar conditions is 16.30%. When the initial pH is 6, DES can be completely degraded within 11 days by JF, but the automatic reduction amount of DES is 34.82%. Moreover, 100 mg/L DES can be degraded by 96.53% in 6 days in LB medium with the initial pH as 7, but the automatic reduction amount of similar condition is 41.17%. When initial pH is 8, strain JF can degrade 98.23% of 100 mg/L DES within 8 days, but the automatic reduction amount of DES in blank LB under similar conditions is 43.39%. When the initial pH is 9, DES can be completely degraded within 7 days, and the automatic reduction amount of DES in a similar condition is 50.65%. Under different initial pH conditions, the degradation rate of DES in LB medium inoculated with JF was considerably higher than that in the blank LB medium. The results indicate that strain JF can degrade DES over a wide range of pH.

### Influence of Culture Temperature on Strain JF Degradability

**FIGURE 6:**
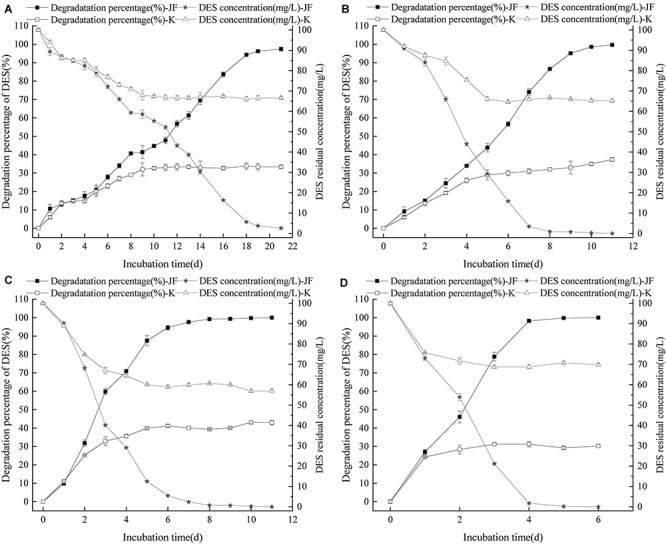
Effect of culture temperature on the DES degradability of JF **(A:** 30°C; **B:** 35°C; **C:** 40°C; **D:** 45°C). K is a control experiment, in which an equal volume of sterile saline solution was inoculated in the DES degradation system instead of strain JF.

Diethylstilbestrol can be degraded effectively by strain JF at different temperatures as presented in Figure 6. Strain JF can degrade DES faster in higher cultivate temperatures; Strain JF degraded 98.46% of DES within 21 days at 30°C, it’s very slowly. Comparably, the DES reduction amount in the blank LB medium under identical conditions was 32.65% in the first 10 days, and then steadily maintained over the extended time. The degradation amount of DES by strain JF at 35°C was considerably higher, with 100 mg/L of DES degraded completely in 11 days at 35°C, and 100 mg/L of DES degraded completely within 6 days at 45°C; the reduction amount of DES in blank LB medium at 45°C was 31.22% in 3 days, which remained steady in the extended period under identical conditions. Strain JF efficiently degraded DES at 45°C as this temperature was most conducive.

### Application Results of Strain JF

In the present study, strain JF could not effectively degrade DES in river water. The result is similar to the results of DES degradation ability of JF inoculated in MSM medium; the residual DES in blank group and treatment group is 1.654 and 1.639 mg/L, respectively. According to the results of this study, DES can be degraded effectively in LB medium rather than MSM medium. The nutrient concentration in the river might be insufficient, leading to the poor growth of JF and ineffective degradation of DES. Thus, further study regarding bio-stimulation could be applied to strain JF by adding extra nutrients through which strain JF can effectively degrade DES.

Strain JF can degrade DES in meat effectively. After being treated for 14 days, the 2 mg/L DES in the meat treatment group was degraded thoroughly. About 32.5% of DES was reduced in the blank group. The results indicate JF can degrade DES in meat effectively, thus providing possibility for JF to treat the DES residue in meat or the DES residue in the waste from the breeding environment of livestock.

### Identification of DES Metabolites

#### Analysis of DES Biodegradation Metabolites by HPLC

**FIGURE 7:**
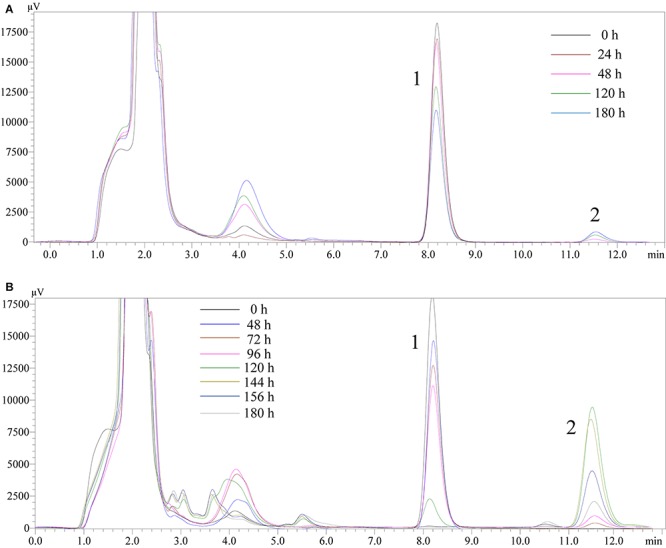
High performance liquid chromatography chromatograms of samples collected from LB-DES culture systems for JF at different incubation time (**A:** LB-DES blank system; **B:** LB-DES-JF strain degradative system).

High performance liquid chromatography detection results (Figure 7) revealed that DES (Chromatographic peak 1) may reduce slightly in the blank LB medium. Peak 2 (retention time, 11.580 min) appeared from 72 h, inferred as a metabolite or a transformed product of DES. In the LB-DES-JF culture systems (Figure 7B), DES was degraded completely at 180 h, and peak 2 was detected at 48 h. Substance 2 accumulated gradually through the degradation process of DES. The concentration of substance 2 reached the maximum value at 120 h, and then it reduced gradually; eventually, it disappeared. In the blank LB-DES culture systems (Figure 7A), the concentration of substance 2 increased to a certain extent with the degradation of DES and then reached an equilibrium value with DES. This indicates that substance 2 was produced by degrading DES, and it can be completely degraded by strain JF but cannot be reduced in the LB-DES system (without strain JF). Other peaks appeared in the chromatograms at different time points in the 3–6 min retention period. As no sign of an antagonistic relationship with DES was observed, these compounds could not be identified as the DES metabolites.

#### Analysis of DES Biodegradation Metabolites by LC-TOF-MS

**FIGURE 8:**
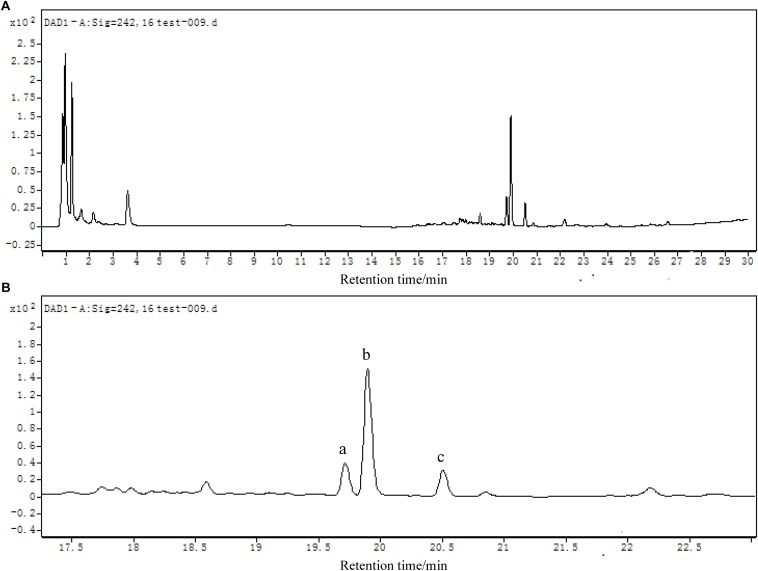
**(A)** LC chromatogram (DAD detector) of mixed samples collected from LB-DES culture systems for strain JF. **(B)** Local amplification chromatogram.

**FIGURE 9:**
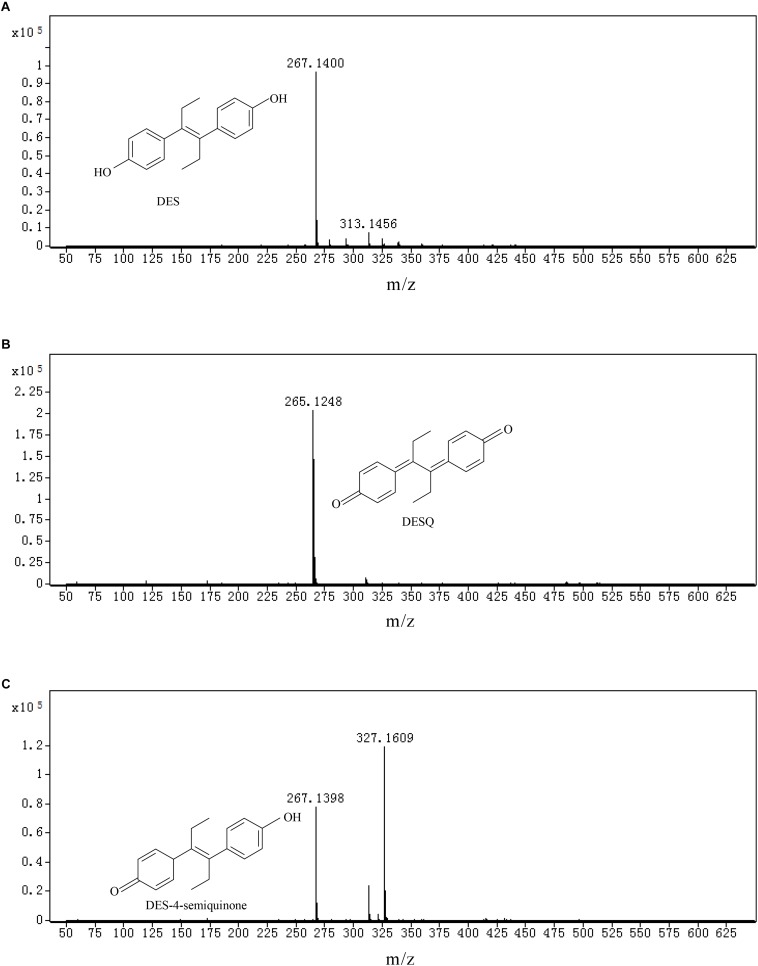
Total iron chromatographs (TIC) and ion fragment plot of mixed samples collected from LB-DES culture systems for strain JF. **(A)** DES, **(B)** DESQ, and **(C)** DES-4-semiquinone.

LC-TOF-MS was performed on the identification of biodegradation metabolites, the molecular structure of each product was deduced by general MS fragmentation rules. Results are depicted in Figure 8 (LC) and Figure 9 (ESI mode MS). The substance with molecular ions at m/z 267.1400 was tentatively identified as DES (Figure 9A). The degrading metabolite with molecular ions at m/z 265.1609, at the retention time of the 20.26 min with a molecular formula of C_18_H_18_O_2_, was proposed to be DES-4, 4′-quinone (DESQ) (Figure 9B). Liehr et al. (1983) reported that DESQ was a metabolite of DES. At the retention time of the 20.85 min, two substances were detected (Figure 9C). One substance’s m/z was 267.1398 (molecular formula: C_18_H_20_O_2_) and the other substance’s m/z was 327.1400 (molecular formula: C_20_H_24_O_4_). The first substance can be proposed to be DES-4-semiquinone, and the second substance was predicted to be [M+CH_3_COOH-H]^–^ (M = DES-4-semiquinone). The results are in accordance with those reported by Zhang et al. (2013). Acetic acid can be produced in the fermentation process but is easy to combine with DES-4-semiquinone due to its instability; thus, both DES-4-semiquinone and [DES-4-semiquinone+CH_3_COOH-H]^–^ can be detected in the fermentation broth. The detection results of LB-DES revealed that both DES and DESQ existed in LB-DES blank control; thus, DESQ was assumed to be a DES conversion intermediate form in blank LB medium without strain JF.

## Discussion

The reports on DES-degrading microbes are limited. In this research, a *B. subtilis* JF with high DES-degrading ability was identified. In order to optimize the conditions for the strain JF to degrade DES and investigate whether strain JF can degrade DES entirely, the degradation characteristics and metabolites of DES were analyzed.

### Strain JF Can Degrade DES Effectively in LB Medium at Different Conditions

To the best of our knowledge, only two DES-degrading microbial strains have been screened previously. *Pseudomonas* sp. J51 was isolated from seawater by Zhang et al. (2013). The strain can degrade 80% of 10 mg/L DES in MSM medium in 1 day. Another microbial strain *Serrati* sp. S was isolated from the activated sludge of a sewage treatment plant by Xu et al. (2014). This strain can degrade 68.3% of 50 mg/L DES in MSM medium within 7 days. *B. subtilis* JF can effectively degrade DES from 25 to 200 mg/L; whereas, 100 mg/L DES can be degraded entirely by the strain JF within 11 days. Thus, this degradation ability of DES by strain JF is considerably higher than the two strains previously published.

Moreover, *B. subtilis* JF can degrade DES efficiently at a wide range of temperatures (30–45°C) and pH (5–9), which indicates that strain JF is able to withstand a variety of adverse environmental conditions, indicating a high feasibility of real application. Our group is performing research on the genes involved in DES degradation by the strain JF, in order to obtain biodegradation of DES through molecular approaches.

### DES Can Be Converted to DESQ in LB Blank but Cannot Be Decomposed Completely

It was confirmed that DES has certain reduction rates in the LB blank medium without the action of strain JF. When JF in blank MSM medium was inoculated with 25 mg/L DES, 10% of DES was reduced within 11 days; this result is in accordance with the study reported by Zhang et al. (2013). The automatic reduction amount of DES in blank LB is higher than that in the blank MSM. This presumably occurs due to the compositions of LB medium, which provide a rich oxidization condition. From the results described previously, the conversion rate of DES in blank LB medium increased while the pH increased. DES was also degraded into DESQ in LB blank medium in absence of strain JF. This may occur due to a dehydrogenation reaction, which can also explain why the DES reduction rate was higher in the alkaline conditions; however, without the action of strain JF, DESQ cannot be further decomposed, thus maintaining equilibrium with DES. These results can further indicate that the reduction of DES in LB blank media without strain JF is a simple conversion phenomenon rather than degradation.

### Analysis of the Intermediate Products and Degrading Pathway of DES Degraded by Strain JF

The studies on the intermediate products produced via biodegradation are rare. DESQ was identified as an intermediate product of DES in the present study (both in LB blank medium and in the degrading system with strain JF), which corroborates the research of Liehr et al. (1983). Liehr et al. (1983) also confirmed that DESQ can consequently damage DNA by integrating with calf thymus DNA without additional enzyme mediation. Thus, DESQ was considered as a carcinogen metabolite of DES. It is imperative to study the degradation method of DESQ. Metzler (1984) investigated and found that DESQ could be transformed into dienestrol, followed by further oxidization due to peroxidase; however, it is not apparent in the present study, as that may be due to the absence of peroxidase-in the test system (LB medium). Indeed, further analysis of DESQ by HPLC revealed that the metabolites can be degraded entirely by *B. subtilis* JF, but will accumulate in the LB blank medium (without strain JF). All of the results demonstrate that the degradation of DES by strain JF can avoid the accumulation of DESQ.

Diethylstilbestrol-4-semiquinone was also identified as an intermediate product of DES degradations by strain JF. Zhang et al. (2013) postulated that the degradation pathway from DES to DES-4-semiquinone is catalyzed by quinoprotein alcohol dehydrogenase. Furthermore, Zhou et al. (2004) studied the photo-oxidation of DES in water with Fe(III) and oxalate as the catalysts. From this study, DES-4-semiquinone and DES-o-catechol are identified as the two pre-dominant intermediates of DES metabolism. Metzler (1984) studied the oxidation of DES by peroxidases from horseradish and mouse uterus in the presence of H_2_O_2_ *in vitro*. In general, DES oxidized into DES-4-semiquinone, which was further oxidized into DESQ. DESQ was cleaved into 4-(4-oxohexan-3-ylidene) cyclohexa-2,5-dienone, and was then further transformed into (z)-4(4-hydroxyphenyl) hex-4-en-3-one, eventually converting into 1-(4-hydroxy-phenyl)-propage-1,2-dione. Based on these researches, DES was initially speculated to be degraded as DES-4-semiquinone, and was further degraded into DESQ with the action of strain JF. The HPLC detection results revealed that DESQ could be degraded entirely by strain JF. Although we did not get the dynamic change of DES-4-semiquinone via HPLC, from the degradability of DESQ by strain JF, DES-4-semiquinone was demonstrated to be degradable by strain JF. No other by-products were detected out by LC-TOF-MS. This demonstrates that DESQ may be degraded into smaller molecules and degraded completely as reported in the previous literature.

**FIGURE 10:**
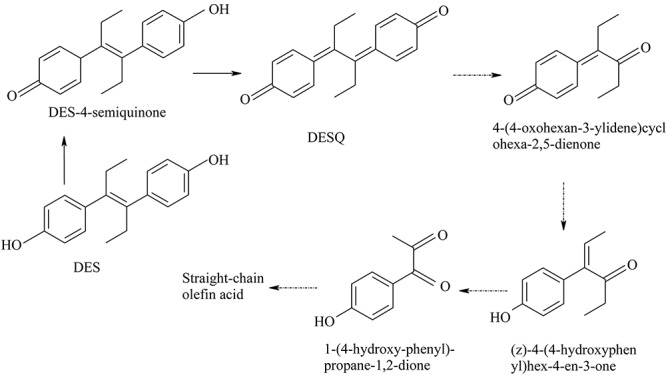
Possible metabolic pathway of degradation of DES by strain JF.

Based on the metabolites identified in the present study and DES oxidative pathways demonstrated in the previous researches, the degradation pathway of DES can be theoretically identified (Figure 10). DES was transformed into DES-4-semiquinone via oxidation and was then further oxidized into DESQ. More oxidation process results in cleavage of the benzene ring decarboxylation that may occur through the mineralization of DES.

## Conclusion

In conclusion, *B. subtilis* JF can degrade 25–200 mg/L DES effectively. The results in this research indicate that higher pH levels lead to higher degradation rates of DES by JF. Temperature also plays an important role in the degradation rate of DES. Temperature of 45°C was confirmed as being optimal for strain JF to degrade DES. Strain JF can degrade 2 mg/L DES in meat thoroughly within 14 days. Two degradation metabolites of DES were identified: DESQ and DES-4-semiquinone, both of which can be degraded completely by strain JF. This study provides novel strain for the reduction or elimination of DES from these contaminated environments. Moreover, the evidence of the microbial metabolic pathway for DES was provided by oxidization and cleavage through the effect of microorganism, which may play an important role in DES biodegradation.

## Data Availability Statement

All datasets generated for this study are included in the article/supplementary material.

## Author Contributions

SL designed the experiments. WD, KH, and YZ performed the experiments. WD, SC, LH, XA, and XH performed the data analysis. YY and LZ provided the scientific expertise. WD wrote the manuscript.

## Conflict of Interest

The authors declare that the research was conducted in the absence of any commercial or financial relationships that could be construed as a potential conflict of interest.
